# The Prevention of Scurvy

**Published:** 1918-01-26

**Authors:** 


### THE PREVENTION OF SCURVY.

### Some Recent Researches upon Vitamines.

CERTAINLY not the least interesting chapter in the annals of bio-chemistry is that which deals with the prevention of such nutritional diseases as scurvy and beri-beri. For long it was recognised that the incidence of these diseases depended largely upon the absence either of fresh food, or, in the case of beri-beri, of a certain "something" from a diet of dried foodstuffs, the nature of which was obscure.

Modern work has confirmed this belief. Beri-beri, as everyone knows, can be practically stamped out by the substitution of unpolished rice (*i.e.*, rice in which some of the germinal layers have been left upon the seed) for the polished rice which often forms the staple diet of the Chinese and East Indian coolie. It was never a disease prevalent among Western peoples, who demanded a menu of varied composition. But even in recent times, and to a limited extent up to the present day, there have been outbreaks of scurvy among those who have been compelled to rely upon preserved and salted foods which in the method of their preservation have been subjected to prolonged heating or drying. Armies in the field, Arctic explorers, and others, whose food has to be carried compactly and in small bulk, suffer from sporadic outbreaks of scurvy, or, if they do not develop the actual symptoms of the disease, show a marked condition of ill-health and inefficiency due to the want of an essential element in their diet.

This something was named "vitamine" by Funk, whose work upon the subject has added materially to our knowledge, and the term has become settled in general use to signify any accessory essential for satisfactory metabolism, and the absence of which from the diet leads to the occurrence of a deficiency disease.

### THE DISTRIBUTION OF ANTI-SCORBUTIC VITAMINES.

It is principally due to recent experimental work conducted at the University of Christiania* and at the Lister Institute,† that it has been satisfactorily established that these vitamines are of two kinds, differing widely from each other in their action and distribution.

That protecting from beri-beri, the anti-neuritic vitamine as it is called, is very widely distributed in every natural foodstuff, being most abundantly found in the seeds of plants—*i.e.*, cereals and pulses. It is not destroyed by drying nor reduced seriously in amount by heating at 100° C. for two hours.

The anti-scorbutic vitamine, on the other hand, is present only in living animal or vegetable tissue. Drying destroys it, and cooking reduces it very appreciably in amount, whilst heating at 120° C. destroys it rapidly. Meat contains far less of this vitamine than does vegetable tissue, and correspondingly larger rations of meat, therefore, must be taken to prevent scurvy. Though absent from dried pulses and cereals, if these are allowed to germinate the anti-scorbutic principle is regenerated with the beginnings of active cell life.

Milk, even when fresh, does not contain a large amount of vitamines, and when "Pasteurised"—*i.e.*, heated to 145° F. for half an hour—they are seriously diminished. Dried preparations of milk are also deficient in anti-scurvy vitamine.

The most valuable sources of vitamines are undoubtedly fresh vegetables and fruit, or the fresh juices expressed from the latter.

### THE DANGERS OF TINNED AND DRIED FOODS.

The importance of these facts, more especially at a time like the present, is not far to seek. Many hundreds of thousands of men are now living under conditions which compel them to subsist almost entirely upon tinned foods. These foods, whilst admirable in variety, yet possess, in virtue of the method of their preparation, little or no vitamines. It is essential, therefore, that these rations should be supplemented by fresh food from outside sources. The most readily transported foods of this order are undoubtedly the potato and the onion, and the anti-scorbutic vitamines they contain are only in part destroyed by cooking. For the prevention of beri-beri any whole-meal flour—*i.e.*, flour containing the germinal element—will suffice. But the problem does not concern only our armies in the field. Owing to the shortage of shipping and of agricultural labour, far less fresh food-stuff is now available for the civilian population than in normal times. For a proportion, certainly, of the working-class population, potatoes are the only readily accessible form of anti-scorbutic vitamine, and a dearth of potatoes, such as was experienced last winter, may well be held accountable for the outbreaks of scurvy in Glasgow, Newcastle, and Manchester.

A movement was on foot a few weeks ago in the daily press to popularise the drying of vegetables and fruits during the autumn for use in the winter and spring. Such products, it was maintained, would be of especial value for dispatch to the prisoners of war now suffering from a restricted diet in enemy countries.

Agreeable as any such addition to the daily dietary would be, it should be clearly understood that these vegetables have, by the process of drying, entirely lost their anti-scorbutic properties.

As long ago as 1720, in the great epidemic which ravaged the Austrian army in Hungary, dried vegetables were found useless for the prevention or cure of scurvy. A similar experience was obtained in the American Civil War, where a large daily ration of dried vegetables, including dried potatoes, failed to prevent the onset of scurvy among the soldiers. Nor can there be any doubt that before the actual onset of the disease there is a period of lassitude and diminished resistance to intercurrent maladies, and that during this period the output

* Axel Holst and others, *Zeitsch. f. Hyg.*, 1912, vol. lxxii.

† Cooper, *Journal of Hygiene*, vol. xiii., 1913, and vol. xiv., 1914; Chick and Hume, *Trans. Soc. Trop. Med. and Hyg.*, vol. x., 1917.

**THE PREVENTION OF SCURVY**—*(continued)*.

of energy by the individual falls far below the normal.

INFANTILE SCURVY.

Owing to the danger from tuberculosis and other milk-borne diseases the substitution for raw milk of boiled or Pasteurised milk is almost universally recommended for the feeding of infants. For the same reason, and on account of the present scarcity of fresh milk, the use of dried milk is constantly on the increase. In this connection a word of warning is very necessary if the health of the coming generation is to be maintained. As a result of experience at the Hebrew Infant Asylum, New York, Hess and Fish* have definitely shown that scurvy developed when infants were fed upon milk "Pasteurised" at 145° F. for thirty minutes. The symptoms, however, speedily cleared up when a little orange juice was given, or if potato water (prepared by adding one tablespoonful of boiled, mashed potato to a pint of water) was added to the milk as a diluent instead of barley water.

There is, we understand, a large amount of dried milk in America at the present time awaiting shipment to this country. In view of a possible shortage of oranges, and the certainty that these will be high in price, it cannot be too widely known that the juices of root vegetables possess, although in a less and varying degree, the same properties in preventing infantile scurvy as orange juice.

* Hess and Fish, *Amer. Journ. of Diseases of Children*, vol. viii., 1914.

